# Grief and Sexual Intimacy: Exploring Therapists’ Views of Bereaved Clients

**DOI:** 10.1080/19317611.2024.2354815

**Published:** 2024-05-20

**Authors:** Sara Jones, Sara Albuquerque, Patrícia M. Pascoal

**Affiliations:** a School of Psychology and Life Sciences, Lusófona University, Lisbon, Portugal; b HEI‐Lab: Digital Human‐Environment Interaction Lab, Lusófona University, Lisbon, Portugal

**Keywords:** Grief, sexual intimacy, couple relationships, bereavement, sexuality

## Abstract

The interaction between sexual intimacy and grief remains unexplored despite its potential to offer valuable insights into how individuals, couples, and society perspectives shape bereaved individuals’ sexual intimacy. Through semi-structured interviews with ten clinical psychologists and psychotherapists specialized in grief therapy, this study explores the impact of grief on sexual intimacy, the challenges faced by bereaved individuals, and the role of sexual intimacy in the grieving process. The study also investigates therapists’ approaches to addressing sexual intimacy within grief therapy sessions. Findings reveal that grief often disrupts sexual intimacy, affecting individuals’ ability to engage emotionally and physically with their partners. Factors such as secondary loss, emotional availability, traumatic experiences, and the nature of the loss contribute to difficulties in resuming sexual intimacy. Nevertheless, the helpful role of sexual intimacy in grief was also highlighted. Therapists note the significance of communication, mutual empathy, and understanding in overcoming these challenges, advocating for therapy to address these issues comprehensively. Moreover, therapist-related, client-related, and shared factors hindering the exploration of sexual intimacy in grief therapy were identified. Strategies for managing these challenges include normalizing discussions around sexual intimacy and death, integrating systemic approaches into therapy, and providing training in sexuality or sexual therapy for grief therapists. Overall, this study underscores the importance of recognizing and addressing the interplay between sexual intimacy and grief to support bereaved individuals effectively. Insights from therapists shed light on potential avenues for enhancing clinical interventions and fostering awareness of the complex dynamics surrounding bereavement and sexuality.

## Introduction

The death of a close person inevitably impacts the bereaved person’s close relationships (Erskine, 2014). The behavior of the bereaved about their intimate relationships may depend on their strategies for coping with grief, which may translate into different combinations of couple dynamics among those who are bereaved. Bereavement refers to the state of experiencing a loss, commonly associated with the death of a loved one. On the other hand, grief is the emotional, physical, cognitive, and behavioral response to that loss (Stroebe et al., 2008). When intimate partners become detached or avoidant after a loss, the additional stress of relational distance or disharmony may aggravate the grief process (Stroebe & Schut, 2016). On the other hand, supportive and validating couple relationships are essential to making meaning from loss (Albuquerque et al., 2017). Within these putative common scenarios, the specific role of sexual intimacy – an essential outcome of a couple’s communication, a component of global intimacy and a proxy for relationship quality (Yoo et al., 2014) – to the grief process needs to be explored in further research. The interaction between death, grieving, sexual activity and sexual intimacy is complex and, even though it affects lives, it may be regarded as a topic that is off limits or societally and culturally unacceptable and, therefore, warrants a deeper discussion.

Despite its relevance, both sexuality and death/grief are often viewed as taboo subjects in many cultures, leading to discomfort or judgment when discussed openly (e.g., Dickson-Swift et al., 2007). This societal reluctance, coupled with the potential for research on bereaved individuals to induce secondary distress among participants, may hinder recruitment efforts, thereby potentially compromising the quality or feasibility of research endeavors. Because this study aims to examine the interaction between sexual intimacy and the grieving process in bereaved individuals, we will approach the topic through the lenses of grief therapy specialists. This will allow us to learn from bereaved clients’ experiences and provide insight into the therapists’ challenges and the best practices they perceive to undertake, allowing us to collect recommendations for other therapists.

For this study, we define sexual intimacy as intimate and consensual sexual experiences (Birnie-Porter & Lydon, 2013) that entail disclosure and perceived partner responsiveness in the context of sex (Rosen et al., 2020). Understanding and promoting sexual health and rights is crucial for overall wellbeing (Sladden et al., 2021) and sexual intimacy in particular is acknowledged to be an essential part of individual wellbeing and couple satisfaction (Skałacka & Gerymski, 2019; Yoo et al., 2014). Therefore, expanding the literature on the sexual intimacy-grief link is relevant because working through grief inevitably involves existing significant relationships (Erskine, 2014).

The Dual Process Model of Coping with Bereavement (Stroebe & Schut, 1999) suggests that individuals adjust to loss through activities, thoughts, and emotions oscillating between loss-oriented coping and the secondary stressors that come about as an indirect consequence of the bereavement, such as changes in identity and roles or mastering new skills (restoration-oriented coping). The focus on interpersonal difficulties, such as the emotional demands of engagement in sexual activity with the partner, may be one example of secondary stressors caused by grief that entail restoration-oriented coping, as couples may need to find new ways to navigate intimacy and address emotional needs as they adjust to life after the loss. Also, disproportionate loss-oriented coping is associated with complex grief and may negatively impact intimate relationships (Stroebe & Schut, 2016). Distance in intimate relationships can cause the loss of its sexual dimension, potentially enhancing isolation and emotional loneliness, which have been associated with lower restoration-oriented coping (Bonanno & Kaltman, 2001).

Grieving can negatively impact social relationships, potentially leading to withdrawal from others and self-isolation (Bonanno & Kaltman, 2001), creating an atmosphere that will make sexual intimacy absent or irrelevant in the early grieving period. Even though sexual intimacy has the potential for meaningful connection between partners, for some, it may be a way of avoiding grief (Hagemeister & Rosenblatt, 1997). Sexual intimacy may, therefore, present as a dyadic component that may act differently across relationships and social groups. For example, risky sexual conduct in response to bereavement was found in LGBT samples (Bristowe et al., 2016). However, deliberately seeking a pleasurable activity or a way to feel better – namely through sexual intimacy – while grieving may bring about feelings of guilt for the bereaved (Fasse & Zech, 2016), prompting bereaved individuals to seek physical distancing and avoid sexual contact, possibly causing anxiety within the couple (e.g., Gewirtz-Meydan & Finzi-Dottan, 2018).

The meaning attached to sexual behavior is influenced by social and cultural norms (Baber & Murray, 2004). Hagemeister and Rosenblatt (1997) study on couples’ sexual relationships after a child’s death found that meanings attributed to sex, the child’s death, and individual grieving are crucial in understanding the absence, decline, or resumption of sexual contact. For example, for some individuals, the idea of sex was “too painful because it was how the child had been made” (Hagemeister & Rosenblatt, 1997, p. 231). Also, parents were repelled by the idea or confused by the sexual desire or lack thereof experienced by their partner. For others, sex was experienced as life-affirming and comforting (Hagemeister & Rosenblatt, 1997).

Despite the existence of some pioneering research on sexuality and bereavement, mainly linked to bereavement due to the loss of a child, the field is characterized by a lack of a solid body of literature that could expand knowledge about the sexual intimacy-grief link and also contribute to a more comprehensive approach to the experiences that couples go through during bereavement. Constraints linked to the sensitivity of both topics (grief/bereavement and sexuality) and how they can trigger negative emotions or embarrassment may be preventing researchers from delving into this topic (Dickson-Swift et al., 2007). Using therapists specialized in grief, that have regular contact with this field, may provide valuable information to overcome the lack of knowledge in this field.

Sexual intimacy is a neglected field in bereavement research due to the sensitivity of the topic, but therapists are well-positioned to give accounts of their bereaved clients’ experiences. Therapists serve as both receivers and producers of knowledge, drawing from their experiences to offer valuable insights into their clients’ experiences, particularly in the context of bereavement. Their expertise positions them as credible sources of information, making their perspectives crucial for understanding and addressing issues like sexual intimacy in therapy (Meuser & Nagel, 2009). Research suggests that collaborative relationships with therapists are essential for positive outcomes in grief therapy, underscoring the significance of studying their views on addressing sexual intimacy to navigate challenges effectively (Klasen et al., 2017). However, despite recognizing the benefits, therapists may feel insecure due to a lack of training in addressing sexuality issues, especially in bereavement contexts (Naughton, 2022). Nonetheless, interviewing experts can yield valuable insights and practices for effectively addressing sexual intimacy in therapy (Træen & Schaller, 2013). Therapists’ influence on therapy outcomes highlights the importance of studying their views and experiences regarding sexual intimacy in bereavement, addressing gaps in research and ethical concerns (Hill et al., 2023).

This study explores grief therapists’ perceptions of the interaction between sexual intimacy and grief. Understanding sexual intimacy’s role in grieving and vice-versa can provide insight into individual, dyadic, and social perspectives shaping bereaved individuals’ sexual intimacy, thus creating awareness and improving clinical interventions for bereaved people. Therefore, we aim to answer two interrelated research questions based on therapists’ perspectives: “Is there an interrelation between sexual intimacy and grief?” and “How can the link between sexual intimacy and grief be approached in therapy?”

## Method

### Participants

The participants for this study were ten clinical psychologists or psychotherapists who were asked to reflect on their clinical experiences with bereaved clients. The recruitment of participants was conducted using purposive and snowball sampling. Professionals were contacted via current professional contacts known to the research team or suggested by previous participants following their interview. The participant age range was between 33 and 58 years. Their clinical experience varied from 10 to 28 years. Seven participants identified as female and three as male. Participants were all licensed clinicians who specialized in grief therapy and, within the last five years, worked with bereaved individuals in private practice, hospice care, or hospitals.

### Procedures

The study received ethical approval from the Scientific Research Ethics and Deontology Committee of the School of Psychology and Life Sciences of Lusófona University in Lisbon, Portugal, and involved semi-structured interviews. Participants were asked about the characteristics of sexual intimacy in their bereaved clients, its role in the grief process, and how sexual intimacy might be experienced in the context of grief. They also discussed how sexual intimacy could contribute to clients’ coping and the most important take-home messages for those experiencing difficulties. Examples of these questions include: After a death, in your experience, what are the perceptions of sexual intimacy of bereaved individuals? In your view, are there changes in sexual intimacy after a meaningful death? What do you perceive to be the main aspects that may negatively and positively influence the sexual intimacy of bereaved persons? In your opinion, could sexual intimacy play a role in terms of the grieving process? If so, in what way?

Even though the core of the interview guide included questions focused on the client’s perspectives, some questions aimed at some processes that occurred in the therapeutic setting: “In your observation of bereaved clients/patients, does the topic of sexual intimacy, defined as engaging in any form of sensual or sexual consensual activity often come up in the consultations? If so, does it happen spontaneously by the patient, or do you usually take the initiative to explore this topic?”.

### Data analysis

The audio recordings were transcribed and coded using Constructivist Grounded Theory, in which theoretical conclusions are drawn based on the data through coding, categorizing, comparative analysis and interpretation (Charmaz, 2006). This method allows for collecting rich data based on participants’ experiences, allowing for an iterative process to construct theory (Chun Tie et al., 2019). The initial phase of line-by-line coding included examining the transcriptions and identifying participants’ descriptions, which were then grouped into categories to integrate and reduce the volume of data. The final phase of axial coding looked at the relationships between the categories and how they could be incorporated into subcategories and broader themes. The first author initially completed the coding process. However, the integrity of codes and themes was regularly discussed in meetings with the research team to reach a consensus and minimize researcher bias. Constantly comparing the codes and categories and referring back to the transcripts helped ensure the validity of the data. Having the interviewer conduct the transcription and coding also helped maintain the data’s integrity. The interview process was considered sufficient once no new codes had begun to arise from the transcriptions.

## Results

The results are organized in two themes: one about the participants’ perceptions of the clients’ experiences of sexual intimacy and grief and another related to the participant’s perceptions of the challenges of sexual intimacy in grief therapy. The theme **Bereaved clients’ experiences of sexual intimacy and grief** aggregates three categories: *Impact of grief on sexual intimacy and influencing factors, Impact of sexual intimacy on grief* and *Restoring sexual intimacy*; the theme **Sexual intimacy challenges in grief therapy** aggregates two categories**:** *Factors that hinder exploration of sexual intimacy* and *Managing sexual intimacy challenges in grief therapy.* Table 1 shows the main results of the iterative coding process and examples of the themes, categories and subcategories found. Participants’ quotations are coded below according to gender and years of clinical experience - e.g., (f, 10) represents a female participant with ten years of clinical experience.

**Table 1.** Main themes and categories.

Themes	Categories	Subcategories
Bereaved clients’ experiences of sexual intimacy and grief	Impact of grief on sexual intimacy and influencing factors	Secondary loss Emotional availability Traumatic and sudden loss Nature of loss The intensity of grief The needs of the bereaved individual Other factors
	Impact of sexual intimacy on grief	Relationship difficulties and disengagement in sexual intimacy Role of sexual intimacy in the recovery process
	Restoring sexual intimacy	Communication: Benefits of communication Role of therapy in communication Other strategies: Recognizing the importance of sexual intimacy Respecting the rhythm and individual needs Searching professional help
Sexual intimacy challenges in grief therapy	Factors that hinder exploration of sexual intimacy	Therapist-related: reluctance, lack of training in sexuality Client-related: lack of sexual intimacy education, being older, and having sensitive history around the topic, the type of loss experienced, and gender Shared: discomfort, the taboo nature of both sexual intimacy and death
	Managing sexual intimacy challenges in grief therapy	Actions to surpass difficulty in sexual intimacy exploration: Explorations of the client’s relationships and sources of support Normalizing discussions around both sexual intimacy and death Building rapport/therapeutic relationship Recognition of benefits for clients
		General actions to integrate sexual intimacy in grief therapy: Assessment of the role of sexuality in client’s lives Normalizing conversations around sexual intimacy and death Importance of systemic approaches Training in sexuality or sex therapy

## Bereaved clients’ experiences of sexual intimacy and grief

### Impact of grief on sexual intimacy and influencing factors

Grief can have an impact on a person’s ability to love and be loved in return, and nine of the therapists said that sexual intimacy is often affected by grief: “sexual intimacy is about connection, and grief is about the loss of connection” (f, 12). Those who experience the most difficulties after a loss likely experience problematic effects in other areas of their life as loss can inhibit the person’s ability to return to many aspects of their lives. The experience of transitional changes, especially in the early period after a loss, are often resolved, but for some, their ability to engage in sexual activity is impaired long-term, or their partners may become distant.

#### Secondary loss

For bereaved individuals, the loss of a relationship or sexual intimacy becomes a second source of grief for those who are already suffering. Sometimes, when a relationship has a particular meaning for the client, and sexual intimacy ceases or stops feeling good, it becomes another loss. For those who live in unhappy relationships, sometimes this feels like a loss that will never end - “when a relationship is bad, or people are just not happy in the relationship, it feels like a loss” (f, 11). For people who experience this secondary loss, they can feel intense loneliness and isolation. It can impact the care and attention given to their intimate relationships and increase feelings of hopelessness.

#### Emotional availability

For some people, sexual intimacy continues to be a positive part of their life while grieving and emotional availability for this intimacy is likely greater in relationships where intimacy is intense and feels secure. For others, grieving can drain their ability to engage at the emotional level they are accustomed to - “it happens, they cannot leave the grieving experience for 10 minutes*”* (m, 25). Emotional availability can be complex and have reverberating effects between the partners, affecting their intimate lives. For many people, emotional availability for sexual intimacy is reduced early in grieving but may be gradually regained.

#### Traumatic and sudden loss

Traumatic or sudden losses may be more likely to cause difficulties with sexual intimacy. The presence of Post Traumatic Stress Disorder or Depression can lead to loss of libido, as can associated medication. There may also be a sensorial element associated with intrusive thoughts and memories, which makes sexual intimacy particularly challenging -“Especially when the loss involves some sort of traumatic experience with strong sensorial stimuli, I would say that in that situation, I would see that this is kind of intrusive” (m, 10). How a death occurs can be relevant to the impact on a person’s sense of safety, which can be important for sexual intimacy. In newer relationships, safe communication and connection may not yet be established and can add to the sense of a lack of physical safety felt after a traumatic loss. Equally, conflict within intimate relationships may leave a person feeling that their intimate partnership is volatile or unsafe. Once a sense of safety within a couple’s relationship has been damaged, recovering can be challenging.

#### Nature of loss

For those who lose their partner, their intimate life as they know it is also taken from them, and the thought of being with someone else can bring excessive feelings of guilt that “can feel like treason or dishonour” (m, 10). For many widowed people, especially those who are older and from religious or culturally conservative backgrounds, losing their partner condemns them to a life without pleasure. Discussing the loss of a spouse or partner with a therapist often increases awareness of all the types and layers of intimacy involved in a long-term relationship, of which sexual intimacy is one dimension.

With the death of a child, the two individual parents face the process of grieving, which can be a complicating factor for their intimate relationship. Participants felt that sexual intimacy often came up in clients who had lost children because relationship issues were so significant. One or both partners often struggle to communicate their feelings and needs with the other partner. There can be a reluctance to reengage with sex because of the link to the conception of the deceased child or because of the fear and uncertainty of becoming pregnant again. For parents, the responsibility of having another child can be overwhelming. If there is anger, blame, or resentment toward the other partner, this is destructive for their relationship and may result in divorce and, in some cases, infidelity. In other clients, participants saw compulsive sexual activity in couples who had lost children. This compulsivity played out both in the desire to have more children quickly but also in avoidance of suffering and comfort seeking: “They want to feel strong emotions” (f, 28).

Parental loss was a subject that arose for some clients in relation to caring responsibilities, which often disproportionately affect women. “I remember some daughters who said they stopped having (sexual) relations because of the pressures of caring” (f, 21). Caring for a dying parent and the aftermath of their loss may cause distress and physical fatigue. Very often, the role reversal of caring for a dying parent left a mark on the lives of their bereaved offspring. There may also be difficulties around privacy when caring for a dying parent, or after losing one parent, the other parent may become the sole focus of their attention as they try to help them overcome their loss. In a few cases, there was a sense that deceased parents could be idealized, and the partner of the bereaved felt negatively evaluated in comparison, which was a source of friction in their relationship. “it can be a kind of jealousy…. like you loved your father more than me” (m, 19). This type of loss was also frequently linked to couples distancing from each other as partners were impatient or insensitive to the bereaved person’s reluctance to engage in sexual intimacy, which sometimes lasted many months and, in some cases, even longer. Notably, four participants highlighted that the closeness of the bereaved person’s relationship to the deceased may be a more significant factor than the formal relationship between them.

#### The intensity of grief

Some individuals may be so consumed by the pain of grief that they have no space for anything else in their lives - “the person can end up completely isolated and just focused on the person they lost” (f, 21). During grieving, clients often struggle to recognize their own needs and to move forward from their sadness. This can cause friction and difficulties in their relationships and prevent them from feeling able to be intimate with their partners.

#### The needs of the bereaved individual

Seven participants noted that sometimes bereaved people place little value on their own needs and may become disassociated from their sense of self and their physical body. Also, they may feel driven to live more as an individual, which distances them from their partner; they may have a strong sense of wanting or not wanting attachment and for some people, the thought of sexual touching brings discomfort, especially in the early stages of grieving. Three participants also mentioned cases where, in the wake of loss, individuals were prompted to reevaluate their lives and relationships, and this could also cause them either to seek intimacy or distance themselves from their partner.

#### Other factors

The age of the deceased and the bereaved were sometimes considered contributory factors. Four participants also mentioned gender as a factor, as they felt that the role and meaning placed upon sexual intimacy differed for men and women. Individual responses to grieving were also highlighted, as well as the acknowledgement that while clients may experience multiple losses in their lives, they may respond to them differently for many reasons. Individuals and couples may also respond differently in times of high anxiety or stress.

### Impact of sexual intimacy on grief

#### Relationship difficulties and disengagement in sexual intimacy

Existing problems in communication within partner relationships were highlighted in all of the interviews as problems, as well as the threat of judgment from a partner. Physical distance from their partner, neglect, guilt, blame and ambivalence are difficulties experienced in intimate relationships, which contribute to greater suffering for bereaved persons. A lack of pleasure in their relationships, perceived disrespect and poor partner understanding, amongst other relational difficulties, were mentioned as potentially impacting sexual or intimate relationships and compounded feelings of loss and emotional distress for those who were already grieving.

Forced or coerced participation in sex was also a frequent topic among female clients in particular. “a wife can feel that a husband is not respecting her, not being emotionally available and forcing sex when she is not in the mood” (m, 19). Often, male partners grew impatient and made demands for sexual intimacy that their grieving partners did not want to fulfill. Resentment and blame on both sides increased the suffering of these clients and became an additional burden. One participant described a particular partner in a client’s life as a “menacing figure” (m, 10).

#### Role of sexual intimacy in the recovery process

Nine participants expressed the idea that sexual intimacy could be helpful. An adaptive bereavement takes place in connection with others through the expression of emotion in order to integrate and process the experience - “it is something that’s shared and the way to cope with it and to wait and to deal with it for me at least, I think that it passes through others” (m, 10).

Three participants acknowledged a slight but meaningful increase in the positive outcomes of those clients who had maintained sexual intimacy during periods of grieving. Most felt that resuming sexual intimacy could also be a sign of recovery. However, the meaning attributed to sexual intimacy for individuals and couples is a relevant aspect to consider before placing too much importance on this factor.

Sexual intimacy as a potential protective factor was suggested as well as being a manifestation of care and a way to validate emotions and, therefore, may be a helpful coping mechanism - “if your sexuality is lived in a relationship where the person has a sense of space, feels empowered and protected and accepted, this is fundamental for the evolution of grief and its integration” (f, 21).

Building trust and safety in the wake of bereavement was also found to be necessary. Sexual intimacy may help build self-esteem and confidence when couples are engaged in it consciously, and positive experiences may be helpful in the re-building of a person’s identity post-grieving and how they project themselves in the future. While touch may be challenging in the early stages for some people, it could be helpful later in the process to reduce rumination and feel less alone. Some clients become dissociated from their physical bodies, and sexual intimacy may also be helpful in this regard.

### Restoring sexual intimacy

#### Communication

For all participants, communication was highlighted as an essential aspect of overcoming difficulties with sexual intimacy while grieving. Mutual empathy and knowledge of the other person were cited as vital for navigating the experience together. Couples should communicate both about their grieving and about their relationship in order to try and understand the other person’s perspective – “I think that communication is one of the most vital ingredients, yeah, and it can be challenging for people, the more that they manage to communicate the better, then the situation will advance” (f, 20). Therapy can help with this process as it may help couples look at alternative ways to communicate, work on misunderstandings, and exchange perspectives between partners. Three participants stated that they liked actively involving partners in homework activities for their clients and encouraged partner communication as a part of the meaning-making process.

#### Other strategies

Therapists highlighted the importance of bereaved individuals’ recognition of the importance of sexual intimacy – “it can be natural, healthy and helpful” (f, 11) and that people should respect their own body and what it needs. Also, there is “enormous wisdom” (f, 21) in your own body, keeping in mind that if you have experienced traumatic losses, there can be layers of trauma that are affected by aspects of sexual intimacy and grieving. A critical aspect highlighted is that each person has their rhythm and should “take the time you need and understand that it is important to respect this” (m, 10). A fundamental message is that one should not feel stigmatized because of traumatic loss and bereavement, and “If you are concerned, then talk to someone; there are professionals who can help, and the more we talk about these difficulties and break taboos, the better” (f, 28).

## Sexual intimacy challenges in grief therapy

### Factors that hinder the exploration of sexual intimacy

These factors can be organized as therapist-related, client-related or shared. Regarding the factors related to the therapist, seven participants were reluctant to approach the topic unless the client specifically brought it up, even though some stated that with the appropriate context, it was a subject they considered essential and were willing to introduce. For eight of the participants in this sample, sexual intimacy was not a topic that frequently arose in their sessions with bereaved clients “when there are difficulties with a couple or when there are losses with symptoms of depression or PTSD, post-traumatic stress, in these cases it happens that this theme comes, maybe with divorce this is one more aspect, it is not very common” (f, 21). Seven participants stated that the initiative for discussing sexual intimacy came from their clients – “when this topic was introduced it left me thinking, how many times have I raised this topic? By my initiative…not many” (f, 21). Lack of training in sexuality on the part of the therapist was also mentioned as a factor that hinders the exploration of sexual intimacy.

The client-related factors included the lack of client education on the topic, client age – older clients were described as more reluctant and younger clients more open – and clients with susceptible history around the topic (e.g., abusive relationships). The type of loss experienced and the gender of the client were also mentioned as aspects that may influence communication on the topic. Shared and more general factors included cultural discomfort and the taboo nature of both sexual intimacy and death.

### Managing sexual intimacy challenges in grief therapy

#### Actions to surpass difficulty in sexual intimacy exploration

Ease with introducing the topic of sexual intimacy was most often linked to explorations of the client’s relationships and sources of support. Familiarity with the topic of sexual intimacy in grieving clients varied among the participants, with four placing great importance on intimate relationships as part of clients’ holistic well-being. The importance of normalizing discussion around both sexual intimacy and death in order to help clients discuss their difficulties was highlighted during the interviews. Also, participants felt that once a rapport was built with the client, there were more potential opportunities for discussing how different aspects of their lives were being affected by their grieving: “It is about, you know, reading a little bit what the other person is bringing to you” (f, 11). Finally, the recognition of benefits for clients of sexual intimacy explorations also promoted therapists’ motivation in addressing issues around sexual intimacy in grief therapy. Therapists perceived that sexual intimacy explorations provided clients with new insights - “usually it ends up being helpful for them to talk about and…I must say there have probably been moments where people do recognize, ‘Well, maybe I never thought about that; it does have an impact’” (f, 11). Also, it helped with recognizing the positive impact of relationships and that the experiences of sexual intimacy with their partner helped them feel loved and cared for in their grieving. For some clients, it was also an opportunity to voice inner conflicts around sex and grief and gain relief from guilt.

#### General actions to integrate sexual intimacy in grief therapy

Some participants advocate that using systemic approaches might be helpful to encourage better communication for clients and their partners and pinpoint that, for example, genograms can be a helpful way to understand both sources of support and the existence of previous losses. Participants also indicate that therapists should try to work with clients about how they see the world, and when intimate relationships are central to their lives, it is crucial to discuss sexual intimacy, so training in sexuality or sexual therapy can help therapists reach an integrative therapeutic approach to bereavement.

## Discussion

This study aimed to explore grief therapists’ perceptions of the interaction between sexual intimacy and grief and present their experiences in approaching and dealing with this topic. The study highlights the bi-directional impact of sexual intimacy and grief.

Regarding the impact of grief on sexual intimacy, participants shared that the pain of grieving may make it difficult for clients to feel they can experience pleasure or disengage from grieving. Also, traumatic loss (and associated symptoms of Post Traumatic Stress Disorder and Depression) and a history of interpersonal trauma can increase difficulties and even cause aversion to touch and body connection (e.g., Strauss et al., 2019). Coherently, sexual function has been related to depression level in the literature (e.g., Bilge et al., 2020). Reconnecting with the physical self can boost agency and focus away from specific memories (Van der Kolk, 2014).

In addition, participants focused on the role of relationship factors like sexual intimacy in grief. For many bereaved people, sexual intimacy may either remain as it was before or resume after some time without significant consequences for their process of grieving. It may become a much-needed refuge and place of comfort and support, which supports existing research on sexual intimacy 's role in relationships and individual well-being (Yoo et al., 2014). Participants in this study felt that their bereaved clients needed to recognize that experiencing pleasure while grieving was not wrong and that sexual intimacy with their partner could be part of restoration-oriented coping as it may help with improving and investing in the intimate relationship or distracting from their grief. The DPM suggests that activities providing time away from loss-oriented coping or restoration-oriented coping activities are beneficial for bereaved individuals (Stroebe & Schut, 1999). However, the extent to which sexual intimacy serves as an effective means of escape from grieving remains a subject of debate (Dyregrov & Dyregrov, 2008), suggesting a potential avenue for future research exploration.

Also, significant relationship difficulties, guilt, fear of public shame, or ongoing trauma may play out within the interaction of sexual intimacy and grief, which can place a heavy load on a person who is already struggling to cope and recover. Participants stated that their clients often experience reduced sexual desire, leading to increased relationship tension, disengagement, and ultimately divorce, especially when partners were not supportive or understanding. This concurs with research highlighting couple distress caused by differences and mismatches in partners’ desire and emotional availability (Dyregrov & Dyregrov, 2008). Relationship and sexual intimacy difficulties may account for stressors and secondary losses that can contribute to overloading grieving individuals, resulting in reduced coping and physical fatigue, potentially increasing the likelihood of complicated grief (Stroebe & Schut, 2016).

In addition, communication was highlighted by all participants as crucial for overcoming relationship difficulties caused by changes in sexual intimacy during grieving. Sexual communication and expression of feelings are essential for sexual satisfaction and function (Mallory, 2022; Pascoal et al., 2019; Sukhanova et al., 2022). However, according to the participants, interventions focused on these skills are often not offered as part of grief therapy. Involving partners in client interventions can help with meaning-making (Hagemeister & Rosenblatt 1997) and increase dialogue around individual needs, which can, in turn, be helpful for the integration of grief (Stroebe & Schut, 1999).

Finally, the difficulty in exploring sexual intimacy is in line with research showing that few bereaved people discuss their sexual intimacy difficulties with therapists, friends, or family (Dyregrov & Dyregrov, 2008). The perceived expectations of others in terms of how grieving should look or how long it should last, as well as the double taboo of discussing sexual intimacy and death, make many bereaved people less open to seeking help. Traumatic or sudden deaths and suicide bereavement may have an even greater social stigma (Chapple et al., 2015). Therapists’ reluctance and lack of ease to talk about sensitive topics may be conveyed implicitly to clients, convincing them that these topics are unimportant or off limits, which may obstruct their discussion (van Deurzen & Arnold-Baker, 2005). Therapists should create a safe space for open dialogue and reflection (Dyregrov & Dyregrov, 2008), allowing clients to address complex topics like sexual intimacy and grief without shame or guilt (Mearns & Cooper, 2018). The need to normalize topics within therapy and move from social and cultural taboos frequently emerged in the interviews. However, as our participants stated, therapists may not be sufficiently prepared to address the topic (e.g., Alarcão et al., 2012; Cesnik & Zerbini, 2017), potentially compromising clients’ ability to disclose their difficulties.

Our results are in line with previous research that consistently demonstrates that therapists feel the need to have more education and training in sexual health and clinical sexology (Abbott et al., 2023). Our participants highlighted that sexual intimacy should be approached with a positive lens (Cruz et al., 2017), i.e., that reckons sexuality as part of the universal human experience and a source of well-being. The reluctance and lack of easiness to address sexual-related issues may be maximized in the context of bereavement. However, it is not an isolated situation, as it reflects most counselors and therapists’ difficulties when addressing this topic (Mollen et al., 2020). Because our participants do not represent all grief therapists and may, due to self-selection bias, be more at ease with this topic, the lack of training and lack of easiness with sexuality issues prevails, which may indicate that it is essential that sexual topics are approached at earlier stages of therapist and clinician training (Timm, 2009), ideally at the undergraduate level, so that it becomes normalized and, complementarily, may pave the way for more profound education at the post-graduate level. Only continuous education from professionals in sexual health will allow clients to benefit from the best standards of care in sexual health. Practitioners, universities, and professional associations should be united and collaboratively promote the best standards of sexual health education across multiple education levels (undergraduate; postgraduate) and multiple contexts (e.g., professional training in grief therapy) to ensure that no political or ideological constraints inhibit the delivery of specialized care when dealing with sexual issues in diverse clinical contexts (Dermer & Bachenberg, 2015). This is in line with Sladden et al. (2021) recommendation of collaborative efforts and comprehensive approaches to create supportive environments to cater to diverse sexual needs across different life stages.

## Limitations

This study’s small sample size may limit its potential. However, it is a good number for such a niche group, especially in a country with low demographics. Also, future research should include interviews with bereaved individuals and therapists to compare experiences. Also, a longitudinal study could better explore the impact on future relationships. Studies on clinical sexology therapists’ perceptions of the sexual intimacy-grief link could be a potential direction for further research. Future studies may expand the international scope of this theme with a more culturally diverse participant sample.

## Strengths of this study and clinical implications

This novel study brought together sexuality and death, therefore contributing to normalizing the discussion of two complex, challenging, understudied, and often taboo topics. One of the strengths of qualitative research is the depth to which a topic can be explored for an unusual topic; thus, it is essential to work in a bottom-up approach such as CGT, where results are based directly on the participants’ words (Charmaz, 2006). By discussing the topic with therapists who have had many years of client experience, it was possible to benefit from their accumulated experience and insight, which aligns with previous research that has collected experiences from therapists to inform clinical practice (Klasen et al., 2017). Also, the current study included several types of losses, such as those of parents, siblings, or close friends, to better understand individuals and their partners in their grieving.

The importance of therapist openness, underpinned by appropriate training and, therefore, the provision of a safe environment for the client to disclose, is a critical factor addressed by this study. During the interview process, it was demonstrated that the more holistic and integrative the approach of the therapist, the greater the openness for discussing sexual intimacy with bereaved people. For this reason, the potential integration of training in sexual intimacy and relationships could be beneficial for grief therapists to broaden the scope of their interventions. Furthermore, the Trauma-Informed Sex Positive approach, introduced by Fava and Fortenberry (2021), highlights the challenge of discussing sexual aspects in one’s life, both for clients and professionals. It emphasizes the importance of employing principles of trauma-informed care to facilitate direct conversations about sex and sexuality. These principles include ensuring safety, building trust and transparency, fostering collaboration and mutuality, and promoting empowerment, choice, and voice. Also of note is the importance of body-based trauma therapies (e.g., Somatic Experiencing) for treating physical and affective trauma symptoms derived from traumatic grief.
